# Medical education at the time of the COVID-19 pandemic

**DOI:** 10.4102/safp.v64i1.5478

**Published:** 2022-02-21

**Authors:** Indiran Govender

**Affiliations:** 1 Department Family Medicine and Primary Health Care, Faculty of Medicine, Sefako Makgatho Health Sciences University of Pretoria, Pretoria, South Africa

**Figure:**
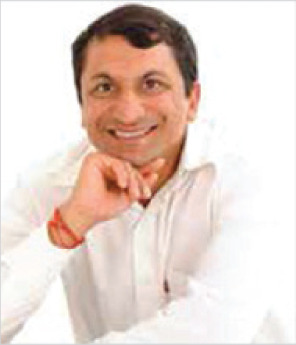


Medical education has been significantly challenged in the current coronavirus disease 19 (COVID-19) era as the Severe acute respiratory syndrome coronavirus 2 (SARS-CoV-2) virus continues to mutate and wreak havoc on society and the global economy. Our social and professional lives have changed as we try to limit the spread of this virus with restrictions of movement, and we explore new ways of doing things. Social distancing, maskwearing, sanitising, zoom meetings, webinars, lockdowns, and other measures have become a daily routine. There have been significant changes fraught with many challenges even on the medical education front. On a positive note, these difficulties have provided new opportunities especially in how we learn and teach.

We have come to understand learners, build partnerships, establish flexible learning environments, understand the new learning climate, how to use the different learning platforms to accommodate students with varied learning preferences and create different contexts of learning. A challenge for online learning is that future doctors require much more than knowledge and skills; the development of compassion and professionalism is an art that cannot be easily taught online.

Teaching undergraduate students has been described as the transmission of authoritative content or the demonstration of procedures. Traditionally, this was the focus of teaching, that is, to provide learners with content, and often more content was provided than was necessary to meet the health needs of a society. The fundamental basis for medical training is to train doctors for the real world. Many medical educators believe that there is no better way than actual hands-on learning under supervision. The concept of bedside teaching with real patients has tremendous benefits in enhancing educational outcomes. This activity is linked to a student’s developmental phase where one is taken through stages from the simple to the more complex as they acquire and develop mastery of more complex skills.

We have long moved away from the traditional world view of behaviourism that described learning as a passive process in response to a stimulus. With cognitivism of the 1960s, the paradigm argued that the mind is a ‘black box’ that should be opened and understood. In the world of medical education, the focus has been on constructivism where new information is constantly added to prior knowledge with the view to mastery of medical knowledge and skills.

Medical schools had to rapidly restructure their curriculum by moving learning to the online platform. Not all experts are comfortable with the new online, virtual approach to medical education. This is especially difficult for older medical educators who feel the onsite mentorship model has worked well for them and should not be discarded. The challenge remains that of building trust and care as well as encouraging a harmonious relationship between teacher and learner.

This pandemic comes at a time where there is a huge availability of technology with the incredible speed of Internet access that ensures content resources are always at one’s fingertips. It is the relevant and appropriate usage of this information that becomes fundamental. An advantage for skills training is the dry run offered by virtual mediums that allow many procedures to be practised before it is performed on real patients such as laparoscopy.

The challenges of online learning must be recognised and dealt with, and this includes cheating with online assessment, the negative social aspects of online learning where participants lose out on the interaction with peers and this isolation has been evident with the increased mental health issues of healthcare professionals, teachers and students. Online learning has motivational problems. The difficulties in teaching and assessing clinical skills online require innovative ways of teaching and assessing these, especially for senior students where workplace-based assessments are necessary now more than ever.

Long before the pandemic, there was concern over the dwindling number of students attending on-site traditional lectures. The majority of students feel that online learning provides greater flexibility, and they either don’t think that online learning has decreased attendance of classes, whereas online teaching has increased student attendance. The other benefits of online learning are less travelling and the possibilities of interacting online wherever you are, decreased travel resulting in decreased pollution and global warming. The older generation of medical educators is concerned about the quality of healthcare professionals produced now with more online teaching, online assessments and video assessments that rely on editing and acting skills, rather than a direct assessment of interactions with patients. The technical aspect of online learning needs to be addressed and is a concern in resource-constrained settings. The resource constraints of many students, medical schools, poorer countries and quality of Internet connections also add to the disproportional learning that may impair already disadvantaged students. The online teaching abilities of lecturers are also a concern for many students, and students in some studies have recommended that medical schools invest and pay attention to upskilling lecturers with regard to online learning and teaching. Students have stated that lecturers develop lectures/lessons that take cognisance of the fact that they are teaching online and keep the students interested.

Medical education seems to be lagging behind the current capabilities regarding online learning. Although medical teachers have doubts regarding the potential of online teaching to produce quality healthcare professionals, students seem to have positive attitudes towards online learning. Students are eager for innovative teaching methods including online learning, networked learning, simulation-based learning, etc. Thus, in a positive manner, COVID-19 has been a catalyst for the inevitable and forced medical education that in many places had nothing online to move to almost a 100% online medical education offering. It seems as we come to terms with COVID-19, a more hybrid nature of medical education is necessary. Students surveyed in many studies suggest a 40% online and 60% on-site ratio of medical education. Most of the medical education community has understood the need for change and has already begun this process. This diffeerent way of providing medical education is here to stay long beyond the COVID-19 pandemic.

